# A Hybrid Multi-Objective Optimization Model for Vibration Tendency Prediction of Hydropower Generators

**DOI:** 10.3390/s19092055

**Published:** 2019-05-02

**Authors:** Kai-Bo Zhou, Jian-Yu Zhang, Yahui Shan, Ming-Feng Ge, Zi-Yue Ge, Guan-Nan Cao

**Affiliations:** 1 MOE Key Laboratory of Image Processing and Intelligence Control, School of Artificial Intelligence and Automation, Huazhong University of Science and Technology, Wuhan 430074, China; zhoukb@hust.edu.cn (K.-B.Z.); zhangjy123@hust.edu.cn (J.-Y.Z.); caognjlu@163.com (G.-N.C.); 2 School of Hydropower and Information Engineering, Huazhong University of Science and Technology, Wuhan 430074, China; 3 School of Mechanical Engineering and Electronic Information, China University of Geosciences, Wuhan 430074, China; fmgabc@163.com (M.-F.G.); zyge@cug.edu.cn (Z.-Y.G.)

**Keywords:** hydropower generator unit, vibration tendency prediction, kernel extreme learning machine, aggregated empirical wavelet transform, Gram–Schmidt orthogonal, multi-objective salp swarm algorithm

## Abstract

The hydropower generator unit (HGU) is a vital piece of equipment for frequency and peaking modulation in the power grid. Its vibration signal contains a wealth of information and status characteristics. Therefore, it is important to predict the vibration tendency of HGUs using collected real-time data, and achieve predictive maintenance as well. In previous studies, most prediction methods have only focused on enhancing the stability or accuracy. However, it is insufficient to consider only one criterion (stability or accuracy) in vibration tendency prediction. In this paper, an intelligence vibration tendency prediction method is proposed to simultaneously achieve strong stability and high accuracy, where vibration signal preprocessing, feature selection and prediction methods are integrated in a multi-objective optimization framework. Firstly, raw sensor signals are decomposed into several modes by empirical wavelet transform (EWT). Subsequently, the refactored modes can be obtained by the sample entropy-based reconstruction strategy. Then, important input features are selected using the Gram-Schmidt orthogonal (GSO) process. Later, the refactored modes are predicted through kernel extreme learning machine (KELM). Finally, the parameters of GSO and KELM are synchronously optimized by the multi-objective salp swarm algorithm. A case study and analysis of the mixed-flow HGU data in China was conducted, and the results show that the proposed model performs better in terms of predicting stability and accuracy.

## 1. Introduction

The role of hydropower stations in the frequency control and peak regulation of the power grid has become increasingly important. Thus, the security and stable operation of the hydropower generator unit (HGU) is crucial to the stability of the power system [1,2]. In practical engineering, the vibration signal is a typical indicator for evaluating the health status and stability of the HGU [3]. Therefore, vibration tendency prediction of the HGU is essential in ensuring safe operation and conducting condition maintenance. It contributes to improving the comprehensive economic benefits of the station. Thus, to guarantee the security of the HGU and avoid economic losses at the station, it is of great importance to study the condition monitoring and tendency prediction of HGUs [4].

Essentially, the vibration tendency prediction of HGU is time series prediction according to historical values. The key aspects for vibration tendency prediction are signal processing and fitting regression [4]. The essential information that reflects the operating states under the environment of noise and electromagnetic interference can be obtained by signal processing [5,6,7]. Then, the vibration tendency of an HGU can be derived by generalized regression. Recently, many advanced methods for tendency forecasting have been applied in engineering, as summarized in Table 1 [4,8,9,10,11,12,13,14,15,16,17]. For instance, Pham et al. proposed a method to forecast the condition of the machine using the auto-regressive moving average (ARMA) [10]. Milovancevic et al. utilized an artificial neural network (ANN) to predict the gear transmission vibration of pellet mills [11]. Fu et al. combined aggregated ensemble empirical mode decomposition (EEMD) with support vector regression (SVR) to predict the vibration tendency of HGUs [12]. Javed et al. applied extreme learning machine (ELM) to forecast the vibration of a turbine engine [13]. In the above methods, ARMA is mainly applied to solve linear problems. However, it is not sufficient to deal with the issue of random fluctuations. Despite the fast calculation of ANN, its performance can be affected by the initial parameters. Moreover, SVR based on structural risk minimization tends to be time-consuming when dealing with constrained optimization issues [18]. Although ELM is widely used for prediction due to its faster convergence speed, considering only the empirical risk minimization principle, it cannot easily obtain the optimal model. In this paper, the kernel extreme learning machine (KELM) is employed as the prediction model, which subtly applies the generalized inverse and least squares to save the computing time [19].

To obtain better prediction results, time-series preprocessing and feature selection methods are also necessary. In term of data processing, short-time Fourier transform (STFT [20]), wavelet transform (WT [21]), empirical mode decomposition (EMD [22]) and its modified methods have been utilized to analyze the vibration data series. In recent years, Gilles proposed a new signal processing method, i.e., empirical wavelet transform (EWT) [23]. It combines the advantages of WT and EMD, which not only has a mathematical theory like WT, but can also decompose signals adaptively like EMD [24]. Hu et al. provided a hybrid model combining EWT with least square support vector machine for wind speed forecasting [25]. The results verified the predictive ability of the hybrid model. Nevertheless, the existing EWT-based forecasting models mainly accumulate all the modes of predicted values. However, there may be some false components among the modes, which will affect the model precision. In addition, it is more time-consuming to predict all the modes. The feature selection is helpful to select the optimal inputs for the prediction model, which improves model accuracy [18,26]. Some methods have been successfully applied in engineering such as partial autocorrelation function (PACF) [27], principal component analysis (PCA) [28] and the Gram–Schmidt orthogonal (GSO) process [26,29].

It has been proven that model optimization can enhance the prediction performance. In general, the objective of model optimization is to obtain the optimal parameters by minimizing the prediction errors [30,31]. For instance, ELM models are optimized by the grey wolf optimizer (GWO) [24] and gravitational search algorithm (GSA) [26]. However, the above models ignore the importance of stability and accuracy, which are equally necessary for evaluating the performance of the prediction model. Unfortunately, these two criteria are almost independent, and can be expressed as multi-objective problems (MOPs) [32]. Single objective optimization algorithms like GWO and GSA will not work. Thus, to handle MOPs, it is necessary to realize the two objectives of good stability and high accuracy simultaneously. In recent decades, driven by engineering practices like water resources scheduling, wind speed forecasting and power load prediction, MOPs have aroused great interest for researchers [33,34]. Pareto optimal solutions can be obtained by multi-objective optimization techniques. Then, they can be presented to the decision maker as a set of feasible optimal options in practice. The multi-objective salp swarm algorithm (MOSSA) is an effective and practical method, which has been widely used in solving MOPs [32,35]. Thus, the MOSSA is introduced to simultaneously realize good stability and high accuracy in this paper.

According to the above discussions, a hybrid model based on AEWT, GSO, KELM and MOSSA is proposed for forecasting vibration tendency. The main contributions are as follows: (1) To eliminate illusive components and reduce the computing time, a mode reconstruction strategy is presented with EWT and sample entropy (SE); (2) MOSSA is applied to find the optimal parameters of GSO and KELM, which can simultaneously obtain good stability and high accuracy of the forecast model; and (3) The hybrid model of AEWT-GSO-KELM in a MOSSA-based framework is introduced for vibration tendency forecasting of HGUs.

The rest of this paper is presented as follows. The framework of the proposed model is illustrated in detail in Section 2, including data preprocess, feature selection, multi-objective optimization, the forecasting and evaluation. Then, Section 3 describes the application results and analysis of the experimental cases. Finally, the conclusions are given in Section 4.

## 2. Proposed Predicting Model and Framework

**Figure 1:**
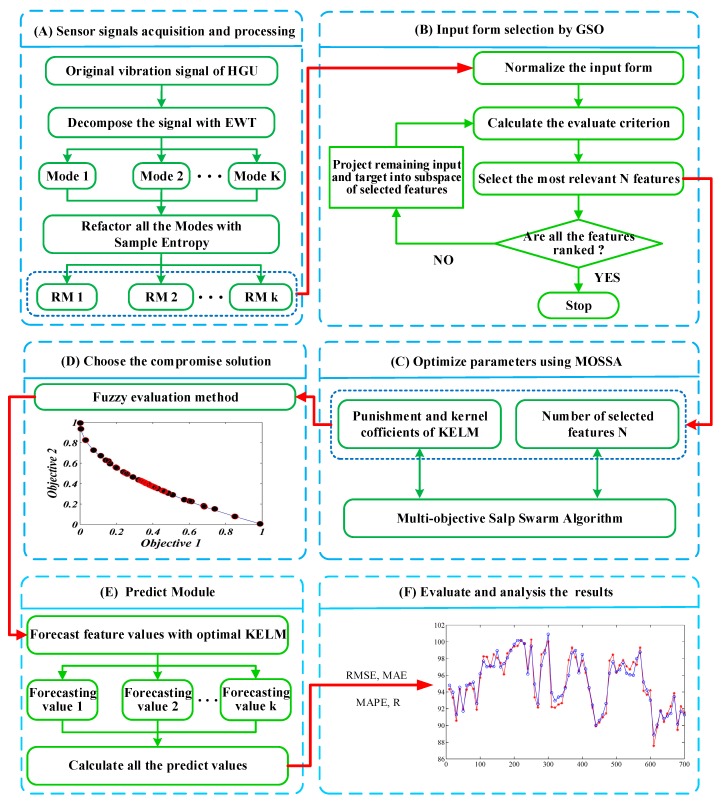
The framework of the proposed model.

To simultaneously obtain higher accuracy and more stable prediction results, a hybrid model is proposed for HGU vibration trend forecasting. Figure 1 shows the basic flow chart. The general procedure contains signal preprocess, features selection, multi-objective optimization, the best compromise solution selection, forecasting and evaluation. The specific procedures are described as follows:

Step 1: Use AEWT to decompose the vibration series into several modes and refactor all modes with the SE-based strategy.

Step 2: Apply GSO method to select the appreciated input features for KELMs.

Step 3: Optimize the parameters N of GSO, C and σ of KELM by MOSSA according to the two objective functions.

Step 4: Use the fuzzy evaluation method to select the compromise solution, i.e., the optimized parameters from the available Pareto front.

Step 5: Establish an optimized KELM prediction model with the optimal parameters of the model and calculate all the forecasting values.

Step 6: Evaluate the final results through several assessment indices quantitatively.

### 2.1. Data Preprocess

The vibration signal is influenced by electromagnetic and environmental factors, which may affect the forecasting result. Thus, it should be preprocessed before the establishment of the forecasting model.

#### 2.1.1. Empirical Wavelet Transform (EWT)

EWT is an adaptive signal processing method [23]. In the EWT framework, the first main step is to divide the frequency domain of the signal according to the Fourier spectrum [36]. Another step is contracting the empirical wavelets, which can extract the AM-FM components with compact Fourier spectrum support [37].

The empirical wavelet function ${\hat{\psi }}_{n}(\omega )$ the empirical scale function ${\hat{\phi }}_{n}(\omega )$ can be respectively determined according to Meyer’s wavelet [38] as follows:

(1) $${\hat{\psi }}_{n}=\left\{ \begin{array}{l} 1\;if(1+\gamma )\omega_{n+1}\leq |\omega |\leq (1-\gamma )\omega_{n}\\ \cos[\frac{\pi }{2}\beta (\frac{1}{2\gamma \omega_{n+1}}(|\omega |-(1-\gamma )\omega_{n+1}))]\;if(1-\gamma )\omega_{n+1}\leq |\omega |\leq (1+\gamma )\omega_{n+1}\\ \sin[\frac{\pi }{2}\beta (\frac{1}{2\gamma \omega_{n}}(|\omega |-(1-\gamma )\omega_{n}))]\;if(1-\gamma )\omega_{n+1}\leq |\omega |\leq (1+\gamma )\omega_{n+1}\\ 0\;otherwise \end{array}\right.,$$

(2) $${\hat{\phi }}_{n}(\omega )=\left\{ \begin{array}{l} 1\;if\left|\omega \right|\leq (1-\gamma )\omega_{n}\\ \cos[\frac{\pi }{2}\beta (\frac{1}{2\gamma \omega_{n}}(|\omega |-(1-\gamma )\omega_{n}))]\;if(1-\gamma )\omega_{n}\leq |\omega |\leq (1+\gamma )\omega_{n}\\ 0\;otherwise \end{array}\right.,$$

where $\gamma \in \min_{n}(\frac{\omega_{n+1}-\omega_{n}}{\omega_{n+1}+\omega_{n}})$ is a coefficient that satisfies the tight frame.

Based on the above, the EWT ($W_{f}^{\varepsilon }(n,t)$) can be constructed as classic WT. The approximation coefficients $W_{f}^{\varepsilon }(0,t)$ and $W_{f}^{\varepsilon }(n,t)$ are illustrated as follows, respectively.

(3) $$\begin{array}{l} W_{f}^{\varepsilon }(0,t)=\langle f,\phi_{1}\rangle \\ W_{f}^{\varepsilon }(n,t)=\langle f,\psi_{n}\rangle \end{array}$$

Finally, the empirical modes *f_k_* are given by:

(4) $$\begin{array}{l} f_{0}(t)=\phi_{1}(t)*W_{x}(0,t)\\ f_{k}(t)=\psi_{k}(t)*W_{x}(k,t) \end{array}$$

#### 2.1.2. AEWT Based on Sample Entropy Theory

The decomposition results of EWT include many modes, some of which are illusive components, and this consumes a lot of time and reduces the forecasting accuracy. To solve the above problems, a mode reconstruction strategy based on sample entropy (SE) is proposed.

According to Richman et al.’s work, the SE is utilized to indicate the randomness of the series [39]. And the SE of obtained modes {x(i) i=1,2,…,j} can be calculated as:

(5) $$P_{SE}(i)=SampEn(x(i),m,r,N),$$

where *P_SE_* represents the sample entropy, and *m*, *r* are the reconstruction dimension and threshold for calculating the SE, respectively.

With the idea of approximate aggregation, the reconstructed mode (RM) can be obtained with the SE values of the modes series *P_SE1_*, *P_SE2_*, …, *P_SEn_*.

(6) $$RM=\sum_{i}^{i+j}Mode_{i},$$

(7) $$s.t.|P_{SE}(n)-P_{SE}(n-i)|\leq \frac{P_{SE}(\max)-P_{SE}(\min)}{n/2}.$$

where *n* represents the number of modes.

Through the reconstruction strategy, the modes will be adaptively divided into groups, where the PE values are closer. The obtained RMs can reduce the illusive components and time consumption. Thus, RMs can be used as input for the proposed model to predict vibration tendency of HGU.

### 2.2. Feature Selection

GSO is a simple and effective method for feature selection, which sorts the correlation between input feature and output target in descending order by a criterion [40]. Suppose the input vector with *k* features of *M* patterns in a system is $X_{k}={\left[x_{k1},x_{k2},\ldots ,x_{kM}\right]}^{T}$ and $Y={\left[y_{1},y_{2},\ldots ,y_{M}\right]}^{T}$ is the corresponding output target. To select the features most relevant to output, the angle between each input $X_{k}$ and output target *Y* can be applied as the criterion:

(8) $$\cos(\varphi_{k})=\frac{\langle X_{k}\cdot Y\rangle }{\left\|X_{k}\right\|\left\|Y\right\|},\;k=1,2,\ldots ,N$$

where $\varphi_{k}$ denotes the angle between the *k*-th input $X_{k}$ and the output target *Y*, *N* represents the number of features. If the input is completely unrelated to output, the $\varphi_{k}$ is π/2. Conversely, $\varphi_{k}$ is 0.

In the iteration process, the features containing the maximum value of the mentioned criterion are chosen as the most relevant feature. For the next iteration, the other candidates and the output vector will be projected into null space of the selected feature. The procedure does not end until all candidate are ranked [41]. Finally, the first *N_f_* features are chosen as the target features. Meanwhile, the number of selected features *N_f_* affects the prediction accuracy and stability.

### 2.3. Kernel Extreme Learning Machine (KELM)

By introducing the kernel function into ELM, KELM gets the least-squares optimal solution, which performs better in generalization [42]. Meanwhile, compared to the traditional single output SVR, KELM can achieve multi-output, which reduces the training time.

The dataset $\left(x_{i},t_{i}\right)$, $x_{i}={\left[x_{i1},x_{i2}\ldots x_{in}\right]}^{T}\in R^{n}$ and $y_{i}={\left[y_{i1},y_{i2}\ldots y_{im}\right]}^{T}\in R^{m}$ represent the input vector and the corresponding output vector, respectively. With the activation function including *L* hidden layer nodes, the output of ELM is calculated as follows [43]:

(9) $$f_{L}(x_{i})=\sum_{j=1}^{L}\beta_{j}h_{j}(x_{i})=h(x)\beta =y_{i}\;j=1,2,\ldots ,N,$$

where β is the output weight vector, and $h(x_{i})$ represents the output of the hidden nodes concerning the inputs $x_{i}$.

The kernel matrix of ELM is obtained by applying the Mercer condition [43]:

(10) $$\begin{array}{l} \Omega_{ELM}=HH^{T}\\ \Omega_{ELM_{i,j}}=h(x_{i})\cdot h(x_{j})=K(X_{i},X_{j}) \end{array}$$

where *H* is the output matrix of the hidden layer:

Then, based on the ridge regression theory, the output function of KELM is formulated:

(11) $$\begin{array}{ll} f(x_{i}) & =h(x_{i})H^{T}{\left(\frac{I}{C}+HH^{T}\right)}^{-1}Y\\ & =\left[\begin{array}{c} K(x,x_{1})\\ K(x,x_{2})\\ \vdots \\ K(x,x_{N}) \end{array}\right](\frac{I}{C}+\Omega_{ELM}){)}^{-1}Y \end{array}$$

The kernel width σ and penalty parameter *C* are both essential for predictive performance. Hence, the best combinations of such parameters should be optimized by advanced intelligent algorithms.

### 2.4. Multi-Objective Optimization

Multi-objective optimization involves multiple objectives, all of which should be optimized simultaneously [44]. In single objective optimization, it is easy to select the best one from a number of solutions through relational operators, while in MOPs, there is more than one criterion, which makes it impossible to compare the solutions. As described in [45,46], Pareto optimal dominance is the main operator that can compare different solutions in MOPs.

#### 2.4.1. Multi-Objective Salp Swarm Algorithm (MOSSA)

MOSSA is an evolutionary computation method based on swarm intelligence. It is inspired by precisely positioning and searching of salps in complex environments [35,47]. With mathematical simulation of the salp chains, they are composed of leaders and followers. Assuming that the food source F is the target of swarm in the search space [32], the leader can update its position according to the food source that is defined as follows:

(12) $$x_{j}^{1}=\left\{ \begin{array}{l} F_{j}+c_{1}((ub_{j}-lb_{j})c_{2}+lb_{j})\;c_{3}\geq 0\\ F_{j}-c_{1}((ub_{j}-lb_{j})c_{2}+lb_{j})\;c_{3}\leq 0 \end{array}\right.,$$

where $x_{j}^{1}$ and $F_{j}$ represent the position of leader salp and the food source in *j*-th dimension, respectively.

The coefficient *c*_1_ can balance exploitation and exploration:

(13) $$c_{1}=2e^{\left(-{\left(4l/L\right)}^{2}\right)},$$

where *L* represents the maximum iteration, and *l* is the current iteration.

The coefficient parameters *c*_2_ and *c*_3_ are random numbers in [0,1].

Then the position of the followers is updated by utilizing Newton’s law of motion:

(14) $$x_{j}^{i}=\frac{v_{final}}{2v_{0}t^{2}}+v_{0}t\;\;i>1,$$

(15) $$v=\frac{\left(x-x_{0}\right)}{t},$$

where $x_{j}^{i}$ is the position of follower salp. $v_{0}$ and *t* are the initial speed and time, respectively.

Since the time is iteration in optimization, considering $v_{0}=0$ and the discrepancy between iterations is equal to 1, Equation (14) is written as follows.

(16) $$x_{j}^{i}=(x_{j}^{i}+x_{j}^{i-1})/2\;\;i>1$$

To explain the MOSSA more clearly and intuitively, the pseudo code is detailed in Algorithm 1.

**Algorithm 1**: MOSSA  ***Parameters:***  *iter_max*—The maximum number of iterations*lb*—Variables lower bound  *obj_no*—Number of objective functions*ub*—Variables upper bound  *dim*—Number of decision variables*N*—The salp population  *archive_maxsize*—The maximum number of archive  /* Set the parameters of MOSSA*/  /* Initialize the salp population $x_{i}(i=1,2,\ldots ,n)$ considering *ub* and lb*/  ***while*** (*iter* < *iter_max*) ***do***  /* Calculate the objective values for each salp *//* Determine the non-dominated salps */  /* Update repository with the obtained non-dominated salps */  ***if*** (the repository becomes full) ***then***  /* Remove the repository resident by calling the repository maintenance procedure*/  /* Add the non-dominated salp to the repository */  ***end if***  /* choose the food source: *F = Select Food (repository)* */  update *c*_1_  ***for*** each salp (*i* = 1 to *N*) ***do***  ***if*** (*i* = = 1) ***then***  update the position of the leading salp  ***else if*** (i≠1) ***then***  update the position of the follower salp  ***end if***  ***end for***  /* Adjuse the salps considering the *ub* and *lb* */  ***end while***
  /* Return repository */

#### 2.4.2. Optimization Strategy

**Figure 2:**
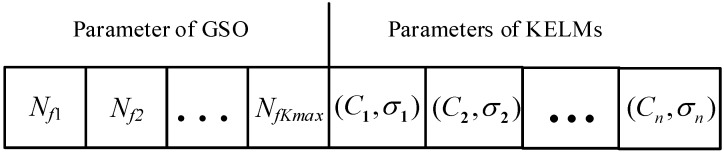
Encoding strategy in the location vector in the multi-objective salp swarm algorithm (MOSSA).

The MOSSA is firstly employed to optimize the parameters of GSO and KELMs simultaneously. As illustrated in Figure 2, the parameters are encoded in the location vector in MOSSA. The first part represents the number of selected features. The remained part is the parameters *C* and σ, which have great influence on the performance of KELM.

#### 2.4.3. The Fitness Function

In this paper, the effectiveness of the prediction model should be simultaneously evaluated by the prediction accuracy and stability. Therefore, the bias-variance indexes are utilized as the fitness functions of MOSSA [30,31]. The value of $Bias(\hat{y})$ represents the precision, which indicates the difference between the true value and the predicted value.

(17) $$Bias(\hat{y})=y-E(\hat{y})$$

where $\hat{y}$ is the predicted value the *n*-th datum, *y* and $E(\hat{y})$ denote the expectation of the observed value and forecasting value, respectively.

Similar to $Bias(\hat{y})$, the $Var(\hat{y})$ indicates the stability of the model. It can represent the variability of the forecast results [48]. The $Var(\hat{y})$ is defined as follows:

(18) $$Var(\hat{y})=E{\left(\hat{y}-E(\hat{y})\right)}^{2}$$

Consequently, the fitness function focusing on high accuracy and stability is formulated as follows:

(19) $$\min\left\{ \begin{array}{l} obf_{1}(x)=Bias(\hat{y})\\ obf_{2}(x)=Var(\hat{y}) \end{array}\right.$$

### 2.5. Evaluation Criterion

To evaluate the performance of different models quantitatively, four evaluated indexes, such as root mean square error (RMSE), mean absolute error (MAE), mean absolute percentage error (MAPE) and coefficient of correlation (R), are employed [18,49].

(20) $$RMSE=\sqrt{\frac{\sum_{i=1}^{N}{\left(y_{i}-{\hat{y}}_{i}\right)}^{2}}{N}}$$

(21) $$MAE=\frac{1}{N}\sum_{I=1}^{N}\left|y_{i}-{\hat{y}}_{i}\right|$$

(22) $$MAPE=100\%\times \frac{1}{N}\sum_{I=1}^{N}\left|\frac{y_{i}-{\hat{y}}_{i}}{y_{i}}\right|$$

(23) $$R=\frac{(1/N)\sum_{i=1}^{N}\left(y_{i}-\bar{y})({\hat{y}}_{i}-\bar{\hat{y}}\right)}{\sqrt{(1/N)\sum_{i=1}^{N}{\left(y_{i}-\bar{y}\right)}^{2}}\times \sqrt{(1/N)\sum_{i=1}^{N}{\left({\hat{y}}_{i}-\bar{\hat{y}}\right)}^{2}}}$$

where *N* represents the sample size. ${\hat{y}}_{i}$ and $y_{i}$ are the predicted value and actual value, respectively.

## 3. Engineering Application and Analysis

**Figure 3:**
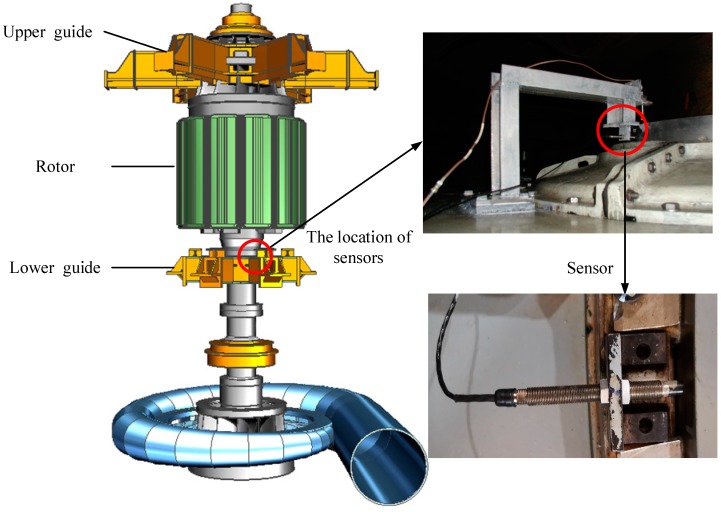
The structure of the hydropower generator unit (HGU) and the location of the sensors.

To verify the effectiveness of the proposed model, the experiment was carried out in the Ertan Hydropower Station in China. The S8000 monitoring system was installed [4], which is made by Shenzhen Strongwish Co., Ltd. (Shenzhen, China). It contains a data acquisition and monitoring substation (NET8000) and central server (WEB8000). The NET8000 acquires the raw signal with sensors and conducts network communication. It can collect up to 4 key phase signals, 24 vibration signals, 12 static signals and 255 process signals obtained by MODBUS communication. The WEB8000 can store and manage the data from NET8000. The general structures of HGU is illustrated in Figure 3, where the location of sensors is shown in on the right. The BENTLY3300 sensors were mounted on the lower guide to obtain the swing data. The sensor is a kind of electric eddy current sensor of the 3300 series, whose output is 4–20 mA or 1–5 V. Its frequency response range is 0–10 kHz and the linear range is 2 mm, average sensitivity is 200 mV/mil [50].

### 3.1. Data Collection

**Figure 4:**
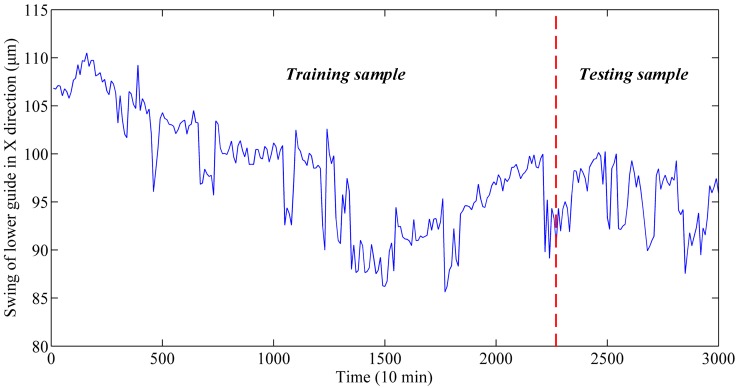
The swing data of lower guide in X-direction.

The complicated structure and frequent switching of operating conditions may easily lead to different monitoring data with different time intervals. Hence, the monitoring data that meets the average time interval was chosen for analysis, which is in line with engineering practice [12]. Thus, the swing data in the X direction from 24 July 2011 to 27 July 2011 were obtained for the experiment. The detailed description of the monitoring data is presented in Table 2. As shown in Figure 4, there are 300 samples with a time interval of 10 min and each sample contains one point. It is obvious that the series has strong non-stationary characteristics. Among all experiments, 3/4 of the entire data were selected randomly as the training set, and the remaining 1/4 were the testing set.

### 3.2. Model Description

To prove the superiority of the proposed model, four types of models were applied and analyzed. The first type includes EEMD-KELM, EWT-KELM and AEWT-KELM, which consist of signal decomposing and model prediction. The second includes AEWT-PCA-KELM and AEWT-GSO-KELM, where PCA and GSO are used for feature selection. The third includes AEWT-GSO-SVR and AEWT-GSO-KELM, where SVR and KELM are utilized to predict the vibration trend. The final type includes AEWT-GSO-SSA-KELM, AEWT-GSO-MOPSO-KELM and AEWT-GSO-MOSSA-KELM.

The above experiments were carried out using the raw data shown in Figure 4. Among the above models, the parameters of the first three models were optimized by MOSSA, while the parameters in the fourth model were optimized by the MOPSO and MOSSA, respectively. The parameters of MOPSO and MOSSA are set as follows: the number of individual agents is 80, the maximum iteration is 100. In addition, for the GSO, the number of selected features is determined in the range [3,12] and the step size is 1.

### 3.3. Vibration Tendency Prediction of the HGU

#### 3.3.1. Vibration Signal Decomposition and Mode Reconstruction

**Figure 5:**
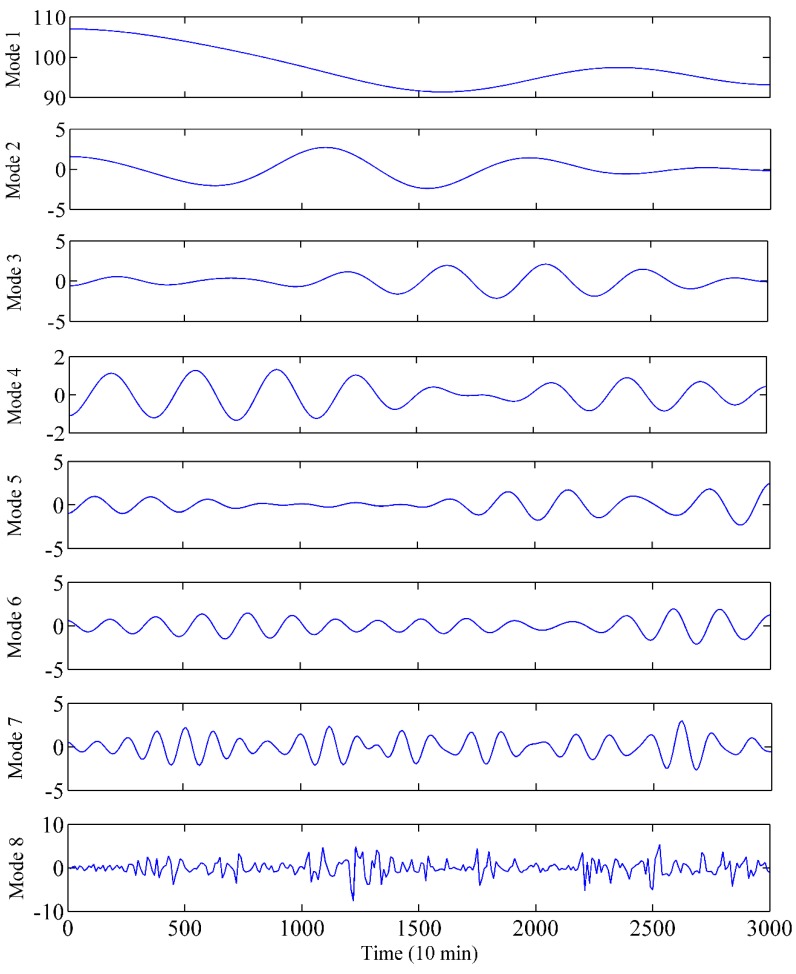
Modes of the vibration data with empirical wavelet transform (EWT).

As shown in Figure 4, the vibration signal fluctuates severely with no clear regularity. Hence, EWT was used to decompose the signal series for decreasing the noise components. The results are shown in Figure 5, where the series is decomposed into several modes.

**Figure 6:**
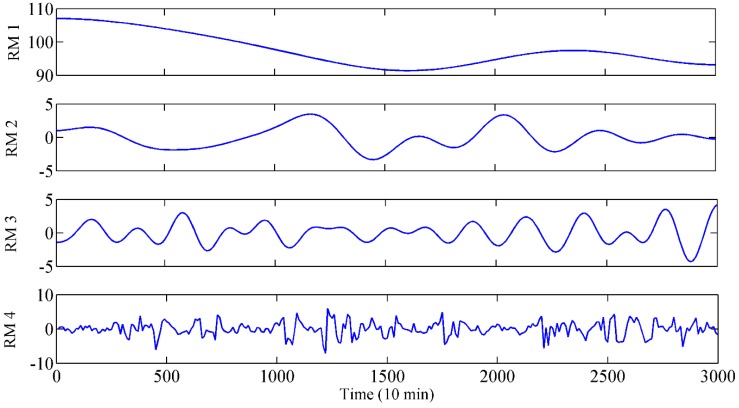
RM obtained from EWT and SE-based reconstruction.

Due to the great impact of IMF complexity on prediction accuracy and computation efficiency of the model, a SE-based IMF reconstruction method was proposed for signal processing. The detailed SE values of the modes are presented in Table 3. It can be seen that the SE values are gradually increasing. This indicates that the complexities of the obtained modes are becoming more and more complex. Based on the proposed reconstruction method, the interval of SE $\frac{H_{s\max}-H_{S\min}}{n/2}$ is set as 0.056. The constructed RMs after mode reconstruction are presented in Table 4. According to the proximity of the SE value, Mode 4, Mode 5 and Mode 6 were selected to construct the RM3. The RM1, RM2 and RM4 were generated by a similar principle. All the obtained RMs are presented in Figure 6, which show significantly different characteristics. More specifically, RM4 shows the strongest nonlinear and the highest frequency, while RM1 is the most stable along the time going. Since the RMs have obvious characteristics and relatively few components, they can be used as inputs of the prediction model to further improve the efficiency and accuracy.

#### 3.3.2. The Selection of the Best Compromise Solution

**Figure 7:**
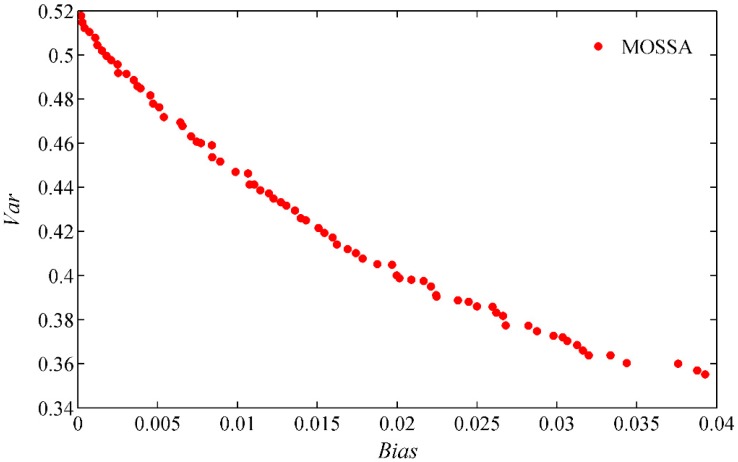
The best solution selection from Pareto-optimal sets of MOSSA.

To avoid randomness and contingency in the optimization, all the experiments were independently repeated 10 times under the same conditions. Therefore, the non-dominated solutions constitute the Pareto front as depicted in Figure 7. It can be seen that MOSSA can easily generate feasible solutions that cover the entire Pareto front. These solutions are distributed with two contradictory objectives. Thus, the fuzzy evaluation method was used to efficiently search for the best compromise solution from the Pareto-optimal front [32,51].

The fitness function of each solution can be obtained with the fuzzy membership function *u_i_*:

(24) $$u_{i}=\frac{f_{i,\max}-f_{i}}{f_{i,\max}-f_{i,\min}},$$

where $f_{i,\max}$ and $f_{i,\min}$ are the maximum and minimum fitness values, respectively. $f_{i}$ is the fitness value of the *i*-th non-dominated solution.

The standardized fuzzy evaluation value *u* can be defined as:

(25) $$u=\sum_{i=1}^{m}u_{i}/m$$

where *m* represents the number of non-dominated solutions.

The maximum value *u* can be chosen as the best compromise solution. In addition, it should be emphasized that the fuzzy evaluation method is only utilized to select the compromise solution, but does not focus on the improvement of the method.

#### 3.3.3. Analysis and Discussion

Based on RMs, the vibration tendency prediction results of the HGU can be obtained by using the above models. The inductors including RMSE, MAE, MAPE and the computing time are utilized to evaluate the performance of the models. Comparing the models further indicates that the integration of reconstruction, feature selection and multi-objective optimization are helpful to improve the prediction accuracy.

**Figure 8:**
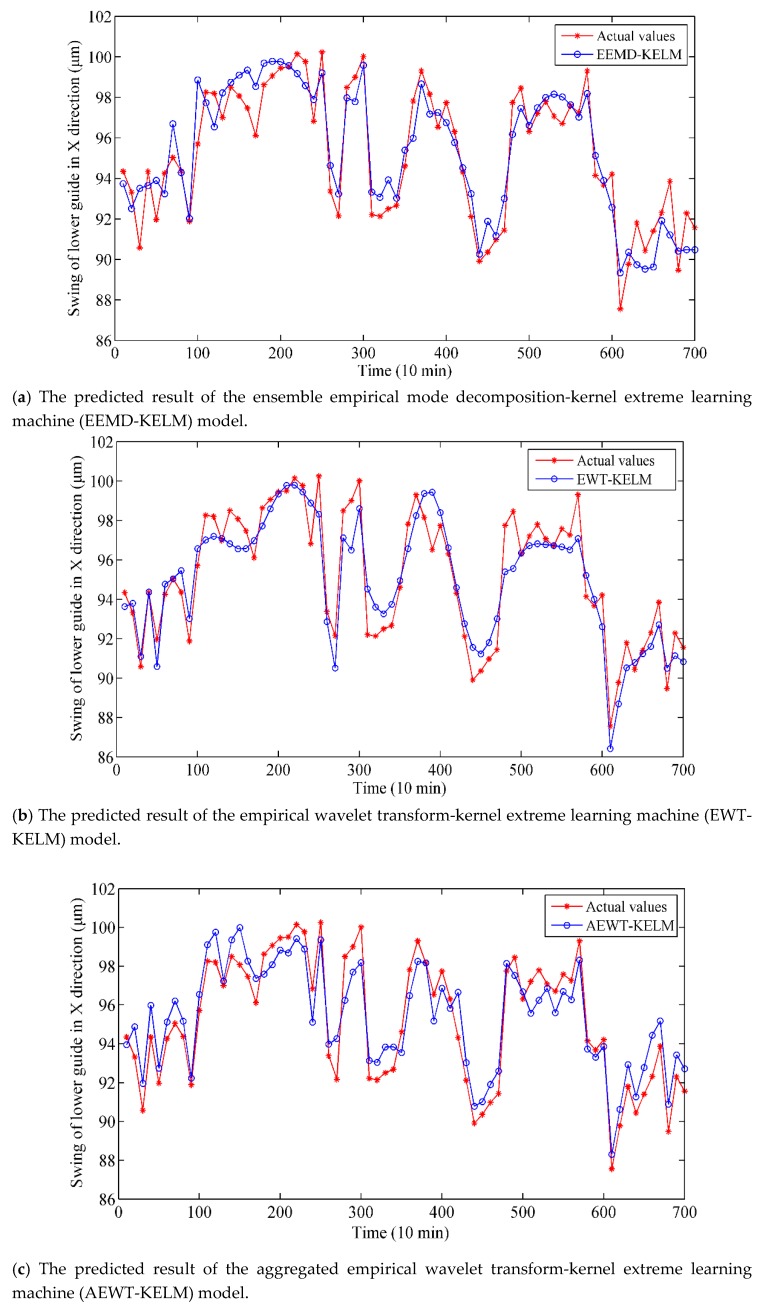

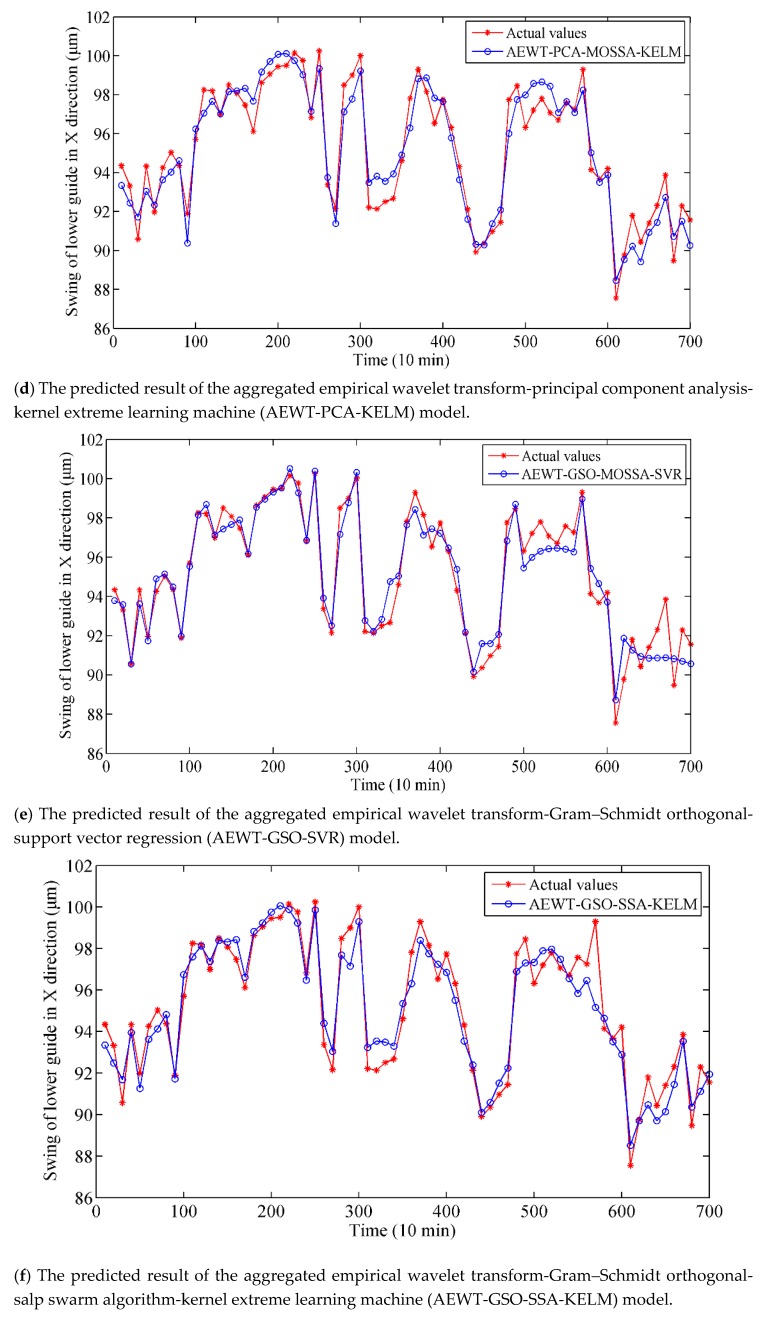

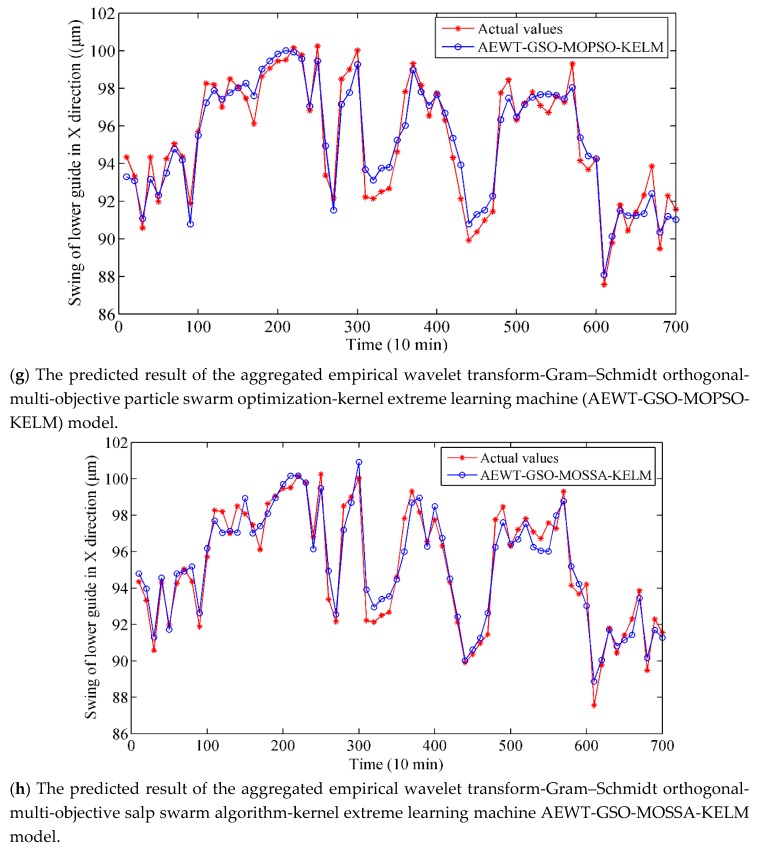
Comparisons of vibration data and prediction results with different methods: (**a**) EEMD-KELM model, (**b**) EWT-KELM model, (**c**) AEWT-KELM model, (**d**) AEWT-PCA-KELM model, (**e**) AEWT-GSO-SVR model, (**f**) AEWT-GSO-SSA-KELM model, (**g**) AEWT-GSO-MOPSO-KELM model, and (**h**) AEWT-GSO-MOSSA-KELM model.

Driven by test data, the predicted vibration trend of the HGU, achieved by eight models is illustrated in Figure 8. The results are worthy of further analysis and discussion.

(1) Compared with the other models of the first type, AEWT-KELM obtains better results than EEMD-KELM and EWT-KELM, which demonstrates that the SE-based reconstruction strategy can improve the forecasting precision.
(2) Among the second type, AEWT-GSO-KELM performs better than AEWT-PCA-KELM, which indicates that GSO is more effective as a feature selection method in these experiments.
(3) By comparing the first and second type models, i.e., AEWT-KELM vs. AEWT-GSO-KELM, we can conclude that the hybrid model integrating the GSO method enhances its performance in forecasting.
(4) Compared with the SVR-based model, the KELM can realize a higher degree of forecasting, which suggests process that the ability of KELM is enhanced by the kernel function.
(5) By comparing the final type models, it can be observed that the proposed model optimized by MOSSA performs better than that by MOPSO, which indicates MOSSA is superior to MOPSO in handling MOPs.

Overall, it is obvious that the prediction curve of the proposed model matches the actual curve best. In contrast, the other models do not fit well with the actual vibration trend. For the SVR-based model, some points of the output are almost divergent in Figure 8e.

**Figure 9:**
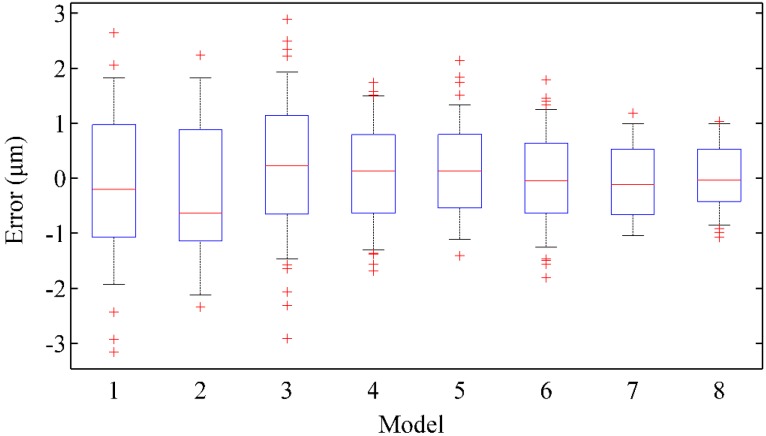
The box plots of error distribution with different models.

The dispersion of the prediction errors is exhibited by box plots as shown in Figure 9, where the eight models are compared. The error index is the relative error between the predicted values and the real ones. The coordinates of the x-axis represent the compared model from EEMD-KELM to the proposed model as ordered in Figure 8. Compared to other models, it can be found that the errors of the proposed model are normally distributed to zero. The errors of the all eight models decrease on tiny scales. This means that the proposed model can achieve uniform precision at almost all points.

To quantify the prediction results, the evaluated indexes of the eight models are illustrated in Table 5. The first three rows represent the results of the first type models. The next four rows are the results of the second and third type models. The remained rows represent the results of the fourth type models, including the proposed model.

From Table 5, the RMSE of EWT-KELM is 1.207, which is lower than that of EEMD-KELM (1.236). The reason is that the EWT method can avoid the mode mixing of EEMD to some extent. Moreover, the RMSE, MAE, MAPE and R of AEWT-KELM are superior to those of EWT-KELM. This shows that the SE-based refactor strategy can obviously enhance the prediction performance and reduce the complexity of the modes. Next, the RMSE of AEWT-GSO-KELM is 0.823, which is lower than that of AEWT-PCA-KLEM (0.919) because GSO can capture the key feature of the series and simplifies the input of KELM. Compared with the SVR-based model, the KELM-based model has better performance, which indicates that KELM has great generalization ability and the over-fitting is reduced by adopting the kernel function. Finally, for MOSSA, the four indexes of the proposed model are 0.823, 0.65, 0.0068 and 0.913 respectively, while the single-objective optimization SSA obtains 0.939, 0.743, 0.0079 and 0.906. This indicates that the performance of the multi-objective optimization algorithm is better than that of single-objective optimization algorithms. Meanwhile, the performance of AEWT-GSO-MOSSA-KELM is superior to that of AEWT-GSO-MOPSO-KELM. For example, the RMSE and MAE of AEWT-GSO-MOSSA-KELM are 0.823 and 0.65, respectively, while those of AEWT-GSO-MOPSO-KELM are 0.841 and 0.731, respectively. This proves that the ability of MOSSA to solve MOP is better than that of MOPSO.

In addition, the correlation coefficient R of the real vibration tendency series and the predicted series by different models are exhibited in the fifth column in Table 5. The results show that the proposed model obtains the highest similarity and correlation with the real series among the different models. In summary, it can be concluded that the proposed model performs best in prediction compared with other models.

Apart from accuracy, efficiency is also a critical goal for model design. Both of these are essential for model evaluation. Thus, the computing time was used to evaluated the efficiency of different models. All the experiments were conducted on the platform with 16 GB of RAM and 3.0 GHz CPU in the MATLAB R2014a environment. The computing time of the nine models in vibration tendency prediction is illustrated in Table 4. As can be seen from the results, the proposed model achieved a better balance between accuracy and efficiency. For example, the time of EWT-GSO-KELM is 105.138 s, which is higher than 97.276 s of AEWT-GSO-KELM. Meanwhile, the RMSE of the former is 1.105, which indicates that the SE-based reconstruction method effectively reduces the computing time and improves the accuracy. In addition, the time of AEWT-GSO-SSA-KELM is 95.716 s, which is lower than that of AEWT-GSO-MOSSA-KELM and AEWT-GSO-MOPSO-KELM. However, its RMSE is 0.939, which is not a satisfactory result. In conclusion, the proposed model achieves a better balance between accuracy and efficiency.

## 4. Conclusions

In this paper, a new vibration tendency prediction model is proposed by integrating SE reconstruction, feature selection and multi-objective optimization. Firstly, raw monitoring data is decomposed into several modes by EWT. The RMs are obtained from all the modes according to the proposed SE reconstruction strategy. Subsequently, the GSO is used to select features for the KELM input for dimensionality reduction. Later, the AEWT-GSO-KELM model is established with the parameters of GSO and KELM optimized by MOSSA, thus simultaneously achieving high accuracy and stability. The proposed AEWT-GSO-MOSSA-KELM model is verified in different comparative experiments, which include the EEMD-based, PCA-based, SVR-based, SSA-based and MOPSO-based models. The results demonstrate that the proposed hybrid model has better comprehensive performance in prediction considering the two objectives of accuracy and stability.

The proposed model was only applied for analyzing data in the existing Ertan station in China. However, if other power stations or other parts of the HGU are to be promoted, some improvements should be made to enhance its applicability and robustness. For example, the fuzzy evaluation method can be improved to select the composited solution. The multi-prediction model will be constructed to predict the data series from different power stations. Therefore, the above-mentioned is also the focus of our subsequent research work.

## Figures and Tables

**Table 1:** Summary of literature on vibration tendency prediction.

Forecasting Models	Methods	Data Set	Authors	Reference
Statistical models	Autoregressive moving average (ARMA) method	The low methane compressor	Pham et al.	[10]
	Grey prediction method	Rolling bearing vibration	Xia et al.	[8]
Artificial intelligence (AI) models	Artificial neural network (ANN)	The gear transmission vibration of pellet mills	Milovancevic et al.	[11]
	Support Vector Regression	The vibration trend of hydro-turbine generating unit	Fu et al.	[12]
	Extreme learning machines	The vibration data of cutting tools and bearing	Javed et al.	[13]
	Long short-term memory recurrent neural networks	The vibration of turbine engine	El Said et al.	[16]
Hybrid models	LS-SVR and chaotic sine cosine algorithm optimization	Vibration trend of hydropower generator	Fu et al.	[4]
	Empirical mode decomposition and relevance vector machine	The vibration signal of bearings	Fei S.-W.	[14]

**Table 2:** Detailed description of the monitoring data.

Sensor	Time	Time Interval	The Number of Samples
BENTLY3300	24–27 July 2011	10 min	300

**Table 3:** The SE values of modes decomposed with EWT.

Indicator	Mode 1	Mode 2	Mode 3	Mode 4	Mode 5	Mode 6	Mode 7	Mode 8
SE	0.0085	0.0381	0.0869	0.129	0.113	0.168	0.212	0.233

**Table 4:** Reconstructed RM derived by EWT according to SE.

RMs	Modes Contained	*H*_S_
1	Mode 1	[0, 0.0085]
2	Mode 2, Mode 3,	[0.0308,0.0869]
3	Mode 4, Mode 5, Mode 6	[0.112, 0.168]
4	Mode 7, Mode 8	[0.177, 0.233]

**Table 5:** The performance of different models.

Model	Precision of Model Prediction				Computing Time
	RMSE (μm)	MAE (μm)	MAPE (%)	R	Time (s)
EEMD-KELM	1.236	1.031	1.093	0.827	106.730
EWT-KELM	1.207	0.997	1.053	0.874	105.138
AEWT-KELM	1.105	1.027	1.157	0.879	98.876
AEWT-PCA-KELM	0.919	0.798	0.846	0.902	100.263
AEWT-GSO-MOSSA-SVR	0.857	0.641	0.681	0.885	109.114
AEWT-GSO-SSA-KELM	0.939	0.743	0.791	0.906	95.716
AEWT-GSO-MOPSO-KELM	0.841	0.704	0.747	0.911	109.987
AEWT-GSO-MOSSA-KELM	0.823	0.650	0.682	0.913	102.920
